# Association between higher intermuscular adipose tissue and decreased renal function in patients with systemic lupus erythematosus mediated by insulin resistance

**DOI:** 10.1186/s13244-024-01722-8

**Published:** 2024-06-18

**Authors:** Bowen Wang, Liping Zuo, Jinlei Fan, Yu Ji, Lei Xu, Min Xu, Yueming An, Yuting Zhang, Guanming Ji, Deixin Yu

**Affiliations:** 1 Department of Radiology, Qilu Hospital of Shandong University, Jinan, Shandong 250012 China; 2 Department of Radiology, The Second Hospital, Cheeloo College of Medicine, Shandong University, Jinan, Shandong 250033 China; 3 Medical Imaging Department, Shengli Oilfield Central Hospital, Dongying, Shandong 257100 China; 4 Department of Radiology, Weihai Municipal Hospital, Cheeloo College of Medicine, Shandong University, Weihai, Shandong 264200 China; 5 Department of Arrhythmia, Weifang People’s Hospital, Weifang, Shandong 261000 China

**Keywords:** Systemic lupus erythematosus, Tomography (X-ray computed), Body composition, Glomerular filtration rate, Insulin resistance

## Abstract

**Objectives:**

To quantify the relationship between abdominal computed tomography (CT)-based body composition parameters and renal function in systemic lupus erythematosus (SLE) patients and evaluate the potential effect of insulin resistance on this relationship.

**Methods:**

SLE patients from institutions A and B between January 2017 and August 2023 were enrolled. Areas and attenuation values of subcutaneous adipose tissue, visceral adipose tissue, intermuscular adipose tissue (IMAT), and skeletal muscle index on CT images were measured at the L3 vertebral level. Logistic regression analysis was used to identify risk factors associated with decreased renal function. Linear regression models were used to describe the relationships between body composition parameters and estimated glomerular filtration rate (eGFR). Finally, we used a single-point insulin sensitivity estimator to indirectly reflect the degree of insulin resistance and assess its mediating effect on the association between IMAT area and decreased renal function.

**Results:**

Three-hundred thirty-nine SLE patients from institution A (internal dataset) and 114 SLE patients from institution B (external validation dataset) were included. Multivariate logistic regression revealed that IMAT area (odds ratio (OR)_institution A_: 1.05 (95% confidence intervals (95% CI): 1.01, 1.10), and OR_institution B_: 1.19 (95% CI: 1.03, 1.39)) was an independent risk factor for decreased renal function in SLE patients. In the adjusted linear regression model, high IMAT area was significantly associated with reduced eGFR (*β*_institution A_ = −1.15, *P*_institution A_ = 0.005; *β*_institution B_ = −0.98, *P*_institution B_ = 0.049). Additionally, insulin resistance contributed a mediating role of 22.8% to the association.

**Conclusion:**

High IMAT area was associated with decreased renal function in SLE patients and insulin resistance mediated this relationship.

**Critical relevance statement:**

High intermuscular adipose tissue area is associated with decreased renal function in systemic lupus erythematosus patients mediated by insulin resistance and is correlated with chronicity index in lupus nephritis patients.

**Key Points:**

- High intramuscular adipose tissue (IMAT) area was associated with decreased renal function in systemic lupus erythematosus (SLE) patients.
- Insulin resistance mediated the association between IMAT area and eGFR.
- IMAT area was associated with chronicity index in lupus nephritis patients.

**Graphical Abstract:**

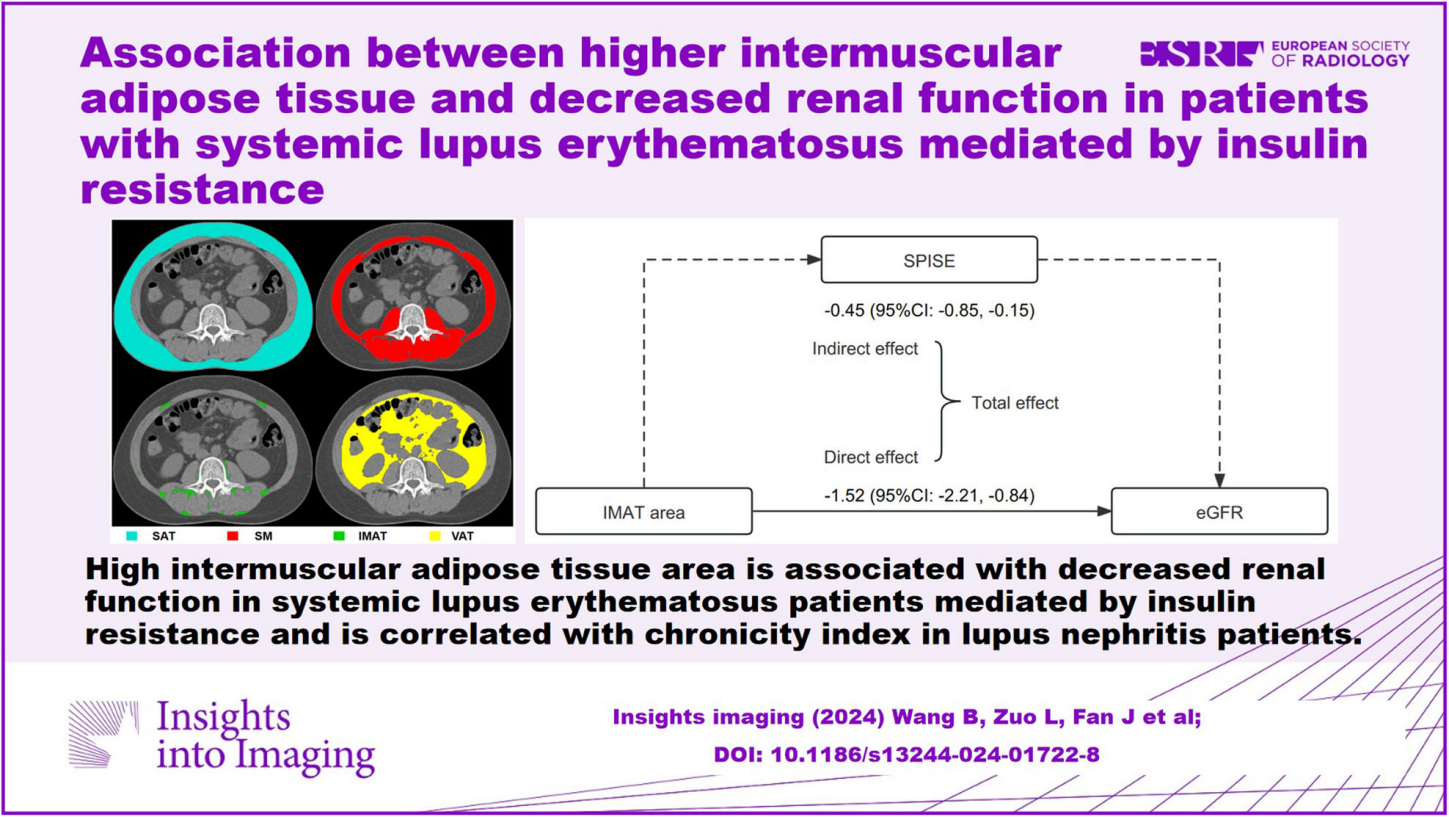

## Introduction

Systemic lupus erythematosus (SLE) is an idiopathic diffuse connective tissue disease with a 2–3-fold higher mortality rate than in the general population [1, 2]. Renal disease is one of the most common and severe manifestations of SLE, with renal injury occurring in approximately 50% of adult patients and 70% of pediatric patients, and is more prevalent in Asian populations [2]. Therefore, identifying the risk factors for SLE-related kidney injury is imperative.

High rates of obesity ranging from 28% to 50% have been reported in SLE patients [3]. Obesity affects the development of SLE through adipokines, which promote the delivery of autoantigens to T cells [4]. Moreover, obesity affects renal function through the recruitment of macrophages by adipokines and the physical compression of the kidneys by fat [5]. In addition, insulin resistance, a central component of metabolic dysregulation in obese patients, is an independent risk factor for the progression of renal disease in humans [6, 7]. Therefore, the importance of quantifying the impact of obesity on SLE-associated kidney injury and identifying obesity-related biomarkers of kidney injury has been increasingly recognized. Although the body mass index (BMI) has traditionally been the most widely used measure of obesity, it cannot distinguish between lean muscles and fat. Muscle biopsy is the gold standard for measuring muscle fat content; however, its invasive nature limits its widespread use. With the development of radiological measurement tools within the last decade, computed tomography (CT)-based scans have facilitated noninvasive assessment of body composition, providing objective measures of fat and muscle composition in patients with SLE [8].

Although previous studies have focused on the adverse effects of a high-fat diet [9], hypertriglyceridemia [10], hypercholesterolemia [11] and BMI [12] on renal function in SLE patients, studies measuring all body components and comparing their associations with renal function in patients with SLE are lacking. Therefore, we aimed to quantify the relationship between body composition measurements based on abdominal CT and renal function in patients with SLE and to analyze the mediating role of insulin sensitivity in the effect on the estimated glomerular filtration rate (eGFR) in patients with SLE, thereby providing recommendations for the early prevention of renal decompensation in patients with SLE and clinical treatment.

## Methods

### Study sample

**Fig. 1:**
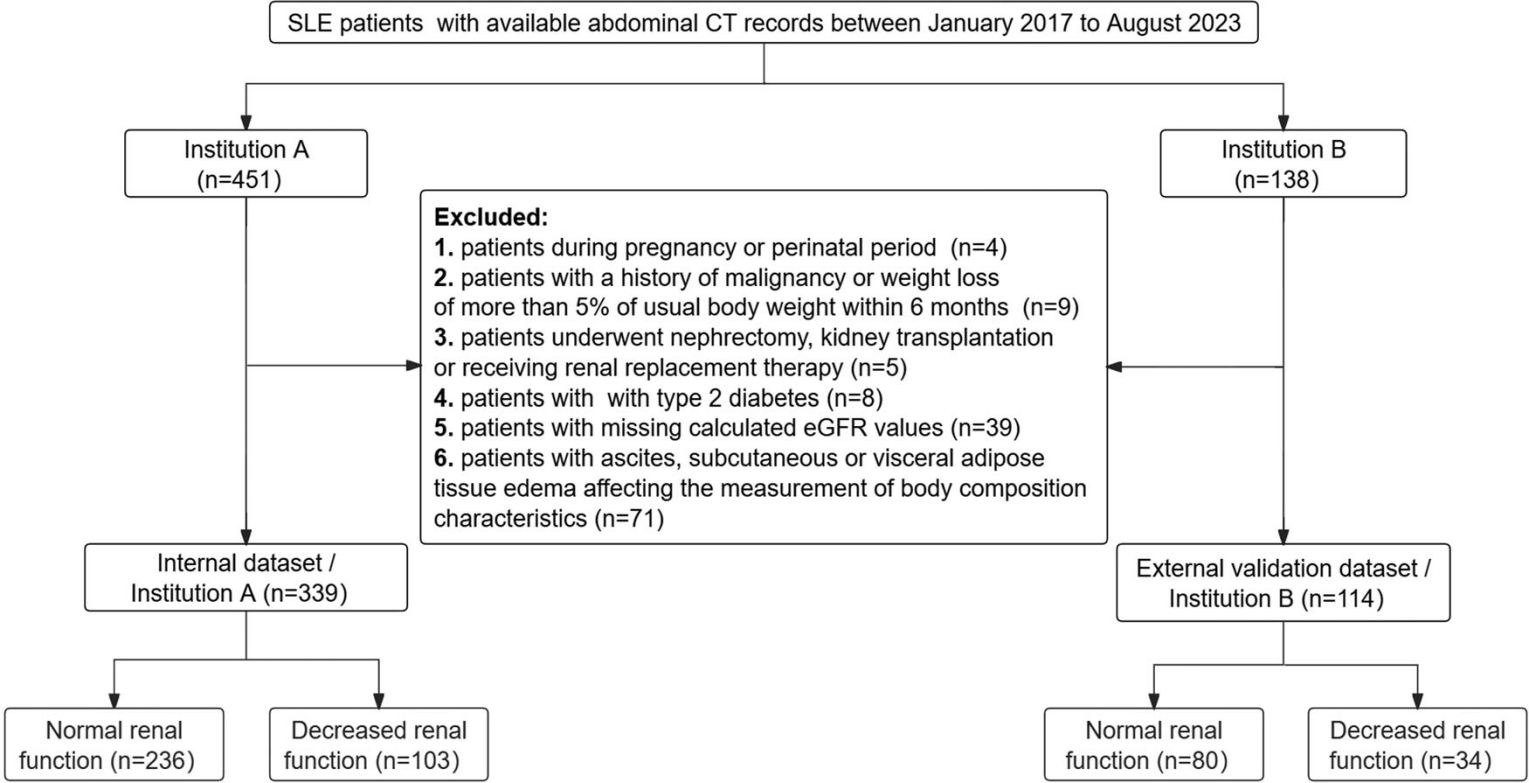
Participant selection flowchart

The electronic medical records of all SLE patients admitted to Qilu Hospital of Shandong University (Institution A) and the Second Hospital of Shandong University (Institution B) between January 2017 and August 2023 were reviewed, and those patients who had undergone abdominal CT scans during admission were enrolled. Patients with the following conditions were excluded: *(a)* women who were pregnant or in the perinatal period; *(b)* history of malignancy or weight loss of more than 5% of usual body weight within 6 months; *(c)* history of nephrectomy or kidney transplantation, or on renal replacement therapy; *(d)* those with type 2 diabetes; *(e)* those with missing values for calculating eGFR; *(f)* those with unmeasured body composition parameters affected by ascites, subcutaneous or visceral adipose tissue (VAT) edema (Fig. 1).

### Clinical data collection and definition

The following demographic characteristics and the first laboratory indicators after the patients’ admission to the hospital of the included patients were obtained from the medical records: age; sex; height; weight; disease duration; glucocorticoid use; hypertension, defined as systolic blood pressure ≥ 140 mmHg, diastolic blood pressure ≥ 90 mmHg, use of any antihypertensive medication, or any self-reported history of diagnosed hypertension; systolic pressure; diastolic pressure; white blood cells; red blood cell (RBC) counts; hemoglobin (HGB) level; red blood cell distribution width; platelets; lymphocytes; neutrophils; monocytes count; erythrocyte sedimentation rate; albumin (ALB) level; albumin/globulin; triglyceride (TG), cholesterol (Cho); high-density lipoprotein cholesterol (HDL-C); low-density lipoprotein cholesterol levels; uric acid (UA); complement C3 (C3); positivity for antinuclear antibodies (ANA), ANA > 1:80 is defined as positive; anti-double-stranded DNA antibodies level; activity index (AI) and chronicity index (CI) in pathology reports of patients with lupus nephritis (LN) from institution A.

The BMI was calculated as weight (kg) divided by the square of height (m^2^). We used a single-point insulin sensitivity estimator (SPISE) index as an index of insulin sensitivity (in inverse relationship to insulin resistance) to indirectly reflect insulin resistance, calculated as follows: SPISE = 600*HDL-C^0.185^/(TG^0.2^ × BMI^1.338^) [13]. The eGFR for children was derived using the Schwartz equation, eGFR (mL/(min/1.73 m^2)^) = *K* × Height × 88.4/Serum creatinine, height in cm, serum creatinine in µmol/L. There are different  *K* values according to different methods of serum creatinine measurement. Since both the organizations involved in this study used Jaffe’s method for serum creatinine determination, we used the   = 0.55 for ages 2–12 years,   = 0.77, and   = 0.55 for male and female patients aged > 12 years, respectively [14, 15]. The adult eGFR values were calculated using the Chronic Kidney Disease Epidemiology Collaboration (CKD-EPI) equation [16]. The most widely accepted eGFR cutoff value for determining decreased renal function remains controversial, and the two most commonly used are < 60 mL/min/1.73 m^2^ and < 90 mL/min/1.73 m^2^, respectively [17–19]. Because the use of higher thresholds is more conducive to the early identification of decreased renal function, 90 mL/min/1.73 m^2^ was adopted in this study as the threshold for decreased renal function. Based on this threshold, all SLE patients were divided into two groups: eGFR < 90 mL/min/1.73 m^2^ (decreased renal function group) and eGFR ≥ 90 mL/min/1.73 m^2^ (normal renal function group).

### CT imaging

Abdominal CT datasets of patients included from the two hospitals were acquired using 64-row spiral CT scanners (Siemens Healthcare Definition AS) from the same manufacturer. The technical parameters for CT imaging were as follows: spiral scanning mode: pitch, 1 mm; tube voltage, 120 kV; tube current, 150 mA; layer pitch, 5 mm; layer thickness, 5 mm; and tube rotation rate: 0.5 s.

### Assessment of abdominal fat and skeletal muscle characteristics

**Fig. 2:**
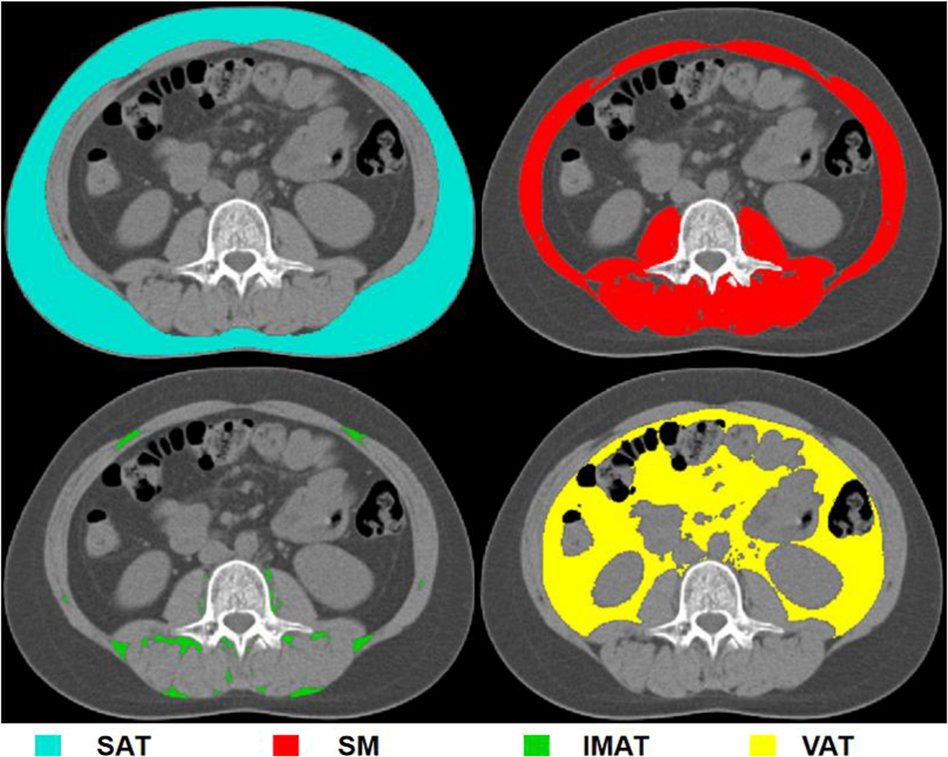
Illustration of body composition measurement from an axial computed tomography slice and a schematic diagram of segmentation. Blue = SAT, Red = SM, Green = IMAT, Yellow = VAT. SAT, subcutaneous adipose tissue; SM, skeletal muscle; IMAT, intermuscular adipose tissue; VAT, visceral adipose tissue

We used non-contrast CT scans and a single cross-sectional axial CT image at the level of the third lumbar (L3) vertebra was analyzed for each patient because this level is currently the most commonly used to assess whole-body body composition parameters (muscle and adipose tissue distributions) [8, 20, 21]. We performed semi-automated segmentation of adipose tissue and skeletal muscle using the Slice-O-Matic software (version 5.0; Tomovision, Montreal, Canada), with the region-of-interest tool used to define thresholds for segmenting fat and muscle. Segmentation was performed independently by a trained operator (B.W.) and reviewed by an abdominal radiologist (D.Y.) with > 30 years of experience, both blinded to the clinical data. The mean of the measured values was obtained. Pixel density in Hounsfield units (HU) was used to identify subcutaneous adipose tissue (SAT) and intermuscular adipose tissue (IMAT) based on a window width of −190 to −30 HU, −150 to −50 HU for VAT, and 29 to 150 HU for skeletal muscle (Fig. 2). Skeletal muscle area (cm^2^) was normalized to height (m) and expressed as the skeletal muscle index (SMI, cm^2^/m^2^). The total adipose tissue (TAT) area was defined as the sum of the SAT and VAT areas, and the VAT/TAT ratio was calculated to explore the distribution of the abdominal adipose tissue.

### Statistical analyses

Continuous variables in clinical and CT data were expressed as mean ± standard deviation and categorical variables were reported as frequencies (proportions). The Kolmogorov–Smirnov test was used to test the normality of the distribution. The independent samples Student’s *t*-test was used for normally distributed continuous variables and the Mann–Whitney *U*-test was used for non-normally distributed continuous variables. Categorical variables comparing the characteristics of SLE patients with normal and decreased renal function were analyzed using the Pearson *χ*^2^ test.

We used SLE patients from institution A as the internal dataset. Univariate and multivariate logistic regression analyses were used to analyze the risk factors for the development of renal impairment in patients with SLE, with the results expressed as odds ratios (ORs) and 95% CI (95% confidence intervals). Spearman analysis was used to explore the correlation between eGFR and clinical and body composition indicators in all patients, and multiple linear regression models were used to describe the dose-response relationship between CT-based body composition parameters and eGFR. To avoid potential multicollinearity and overfitting, we excluded parameters with Spearman’s or Pearson correlation coefficients > 0.7 between the body composition parameters. To verify the reproducibility of the relationship between IMAT and eGFR in SLE patients, we repeated the above statistical analyses in SLE patients from institution B for independent external validation.

Next, we analyzed the association between SPISE and reduced eGFR and adjusted for the IMAT area in Model 3 to explore whether this association was independent of the IMAT area. Considering the association between IMAT area, SPISE, and reduced eGFR, we performed mediation analyses to further explore whether insulin resistance mediates the relationship between IMAT area and reduced eGFR in SLE patients. Subsequently, we performed a subgroup analysis of LN patients diagnosed by renal biopsy to explore the correlation between AI, CI, clinical indicators, and body composition parameters, and further explored the relationship between body composition parameters and CI in LN patients using multiple linear regression.

The sample size for this study was determined based on the original study plan and was 5, 10, or 20 times the number of variables in the multivariate logistic regression analysis model to minimize the risk of overfitting [22]. Statistical analyses and graph construction were performed using the statistical software SPSS 26.0 for Windows (IBM Corp., Armonk, NY, USA) and GraphPad Prism 9.5.1 (La Jolla, CA, USA). Statistical significance was set at *p* < 0.05.

This retrospective study was approved by the Research Ethics Committee (KYLL-202307-043). The requirement for informed consent was waived owing to the retrospective nature of the study.

## Results

### Demographic and clinical characteristics of the study sample

Among 589 SLE patients with available non-contrast abdominal CT scans between January 2017 and August 2023, we excluded patients who were pregnant or perinatal (*n* = 4), had a history of malignancy or weight loss of more than 5% of usual body weight within 6 months (*n* = 9), had undergone nephrectomy, kidney transplantation or renal replacement therapy (*n* = 5), patients with type 2 diabetes (*n* = 8), patients with missing calculated eGFR values (*n* = 39), and patients with ascites, subcutaneous or visceral adipose tissue edema affecting the measurement of body composition characteristics (*n* = 71) (Fig. 1). Of the 589 patients, 453 SLE patients were included in our analyses. 339 of them were from institution A and 114 from institution B.

Of the 339 SLE patients from institution A, the mean age and BMI of the patients were 37.26 ± 16.55 years and 22.39 ± 4.61 kg/m^2^, respectively. The mean disease duration was 4.04 ± 5.95 years. Based on the eGFR values, the patients were divided into two groups: the normal renal function group with eGFR ≥ 90 mL/min/1.73 m^2^ (*n* = 236 [69.6%]) and the decreased renal function group with eGFR < 90 mL/min/1.73 m^2^ (*n* = 103 [33.4%]). Of the 103 patients in the decreased renal function group, 42 had undergone a renal puncture biopsy. Age (34.53 ± 16.34 vs. 43.50 ± 15.37, *p* < 0.001), weight (57.25 ± 14.66 vs. 61.50 ± 13.37, *p* = 0.016), and BMI (21.95 ± 4.56 vs. 23.36 ± 4.59, *p* = 0.005) were lower in patients with normal renal function than in patients with decreased renal function. Hypertension was less prevalent in patients with normal renal function (31/236 [13.1%]) than in those with decreased renal function (52/103 [50.5%]) (*p* < 0.001). In contrast, SPISE was higher in those with normal renal function than in those with decreased renal function (9.73 ± 3.23 vs. 8.39 ± 2.22, *p* < 0.001). No significant differences in sex (*p* = 0.576), height (*p* = 0.807), disease duration (*p* = 0.348), or glucocorticoid use (*p* = 0.089) were observed between the two groups. The other baseline characteristics are summarized in Table 1. The baseline characteristics of 42 SLE patients with available renal puncture results from institution A were presented in Supplementary Table S1. Detailed baseline information of 114 SLE patients from institution B were shown in Supplementary Table S2 and Supplementary Table S3.

**Table 1:** Baseline characteristics and body composition parameters of systemic lupus erythematosus (SLE) patients from institution A according to renal function

Variables	Total (*n* = 339)	eGFR ≥ 90 (*n* = 236)	eGFR < 90 (*n* = 103)	*p*
Age, years	37.26 ± 16.55	34.53 ± 16.34	43.50 ± 15.37	< 0.001
Sex				0.576
Male, *n* (%)	41 (12.1)	27 (11.4)	14 (13.6)	
Female, *n* (%)	298 (87.9)	209 (88.6)	89 (86.4)	
Height, cm	161.30 ± 9.53	160.99 ± 10.40	161.98 ± 7.19	0.807
Weight, kg	58.58 ± 14.38	57.25 ± 14.66	61.50 ± 13.37	0.016
BMI, kg/m^2^	22.39 ± 4.61	21.95 ± 4.56	23.36 ± 4.59	0.005
Disease duration, years	4.04 ± 5.95	3.53 ± 5.22	5.23 ± 7.24	0.348
Glucocorticoid use, *n* (%)	221 (65.2)	147 (62.3)	74 (71.8)	0.089
Hypertension, *n* (%)	83 (24.5)	31 (13.1)	52 (50.5)	< 0.001
Systolic blood pressure, mmHg	127.77 ± 23.16	121.56 ± 18.84	142.00 ± 25.80	< 0.001
Diastolic blood pressure, mmHg	79.29 ± 14.97	76.36 ± 12.88	86.03 ± 17.16	< 0.001
WBC, 10^9^g/L	6.49 ± 4.55	6.27 ± 4.51	6.99 ± 4.64	0.063
RBC, 10^9^g/L	3.72 ± 0.77	3.88 ± 0.72	3.36 ± 0.75	< 0.001
HGB, g/L	107.42 ± 22.66	111.31 ± 21.30	98.45 ± 23.24	< 0.001
RDW, %	14.76 ± 3.14	14.88 ± 3.42	14.51 ± 2.35	0.711
PLT, 10^9^g/L	194.49 ± 110.35	197.43 ± 114.62	187.69 ± 100.02	0.529
LYM, 10^9^g/L	1.49 ± 2.94	1.30 ± 0.87	1.92 ± 5.20	0.603
NEU, 10^9^g/L	4.97 ± 5.98	4.38 ± 3.75	6.33 ± 9.19	0.011
MON, 10^9^g/L	0.51 ± 0.81	0.50 ± 0.82	0.51 ± 0.79	0.389
ESR, mm/h	46.40 ± 36.28	45.22 ± 36.15	49.32 ± 36.63	0.258
ALB, g/L	34.87 ± 7.19	35.99 ± 7.09	32.29 ± 6.78	< 0.001
A/G	1.25 ± 0.40	1.23 ± 0.38	1.31 ± 0.44	0.150
TG, mmol/L	2.14 ± 7.58	2.13 ± 9.04	2.17 ± 1.54	< 0.001
Cho, mmol/L	4.73 ± 2.35	4.48 ± 1.59	5.32 ± 3.48	0.007
HDL-C, mmol/L	1.16 ± 0.45	1.16 ± 0.46	1.16 ± 0.43	0.862
LDL-C, mmol/L	2.65 ± 1.20	2.59 ± 1.19	2.82 ± 1.20	0.178
eGFR, mL/min/1.73 m^2^	103.15 ± 44.07	125.71 ± 28.57	51.44 ± 26.05	< 0.001
UA, umol/L	322.28 ± 183.75	273.16 ± 182.41	433.70 ± 131.10	< 0.001
C3, g/L	0.78 ± 1.75	0.67 ± 0.58	1.00 ± 2.97	0.005
ANA, *n* (%)	301 (88.8)	213 (90.3)	88 (85.4)	0.043
anti-dsDNA, IU/mL	317.19 ± 430.75	316.86 ± 413.92	317.96 ± 470.47	0.201
SPISE	9.32 ± 3.02	9.73 ± 3.23	8.39 ± 2.22	< 0.001
TAT area, cm^2^	216.51 ± 137.03	206.16 ± 138.27	240.22 ± 131.74	0.006
VAT/TAT ratio, %	37.40 ± 12.59	35.32 ± 12.46	42.18 ± 11.61	< 0.001
SAT area, cm^2^	132.89 ± 86.64	130.53 ± 87.07	138.28 ± 85.84	0.312
SAT attenuation value, HU	−93.84 ± 14.16	−95.34 ± 13.69	−90.40 ± 14.66	0.002
VAT area, cm^2^	83.62 ± 61.95	75.63 ± 61.22	101.94 ± 59.98	< 0.001
VAT attenuation value, HU	−81.81 ± 13.54	−81.41 ± 13.60	−82.73 ± 13.43	0.403
IMAT area, cm^2^	9.30 ± 7.59	8.41 ± 6.56	11.34 ± 9.26	< 0.001
IMAT attenuation value, HU	−60.40 ± 7.87	−60.36 ± 8.16	−60.51 ± 7.21	0.871
SMI, cm^2^/m^2^	38.54 ± 8.68	38.12 ± 8.79	39.48 ± 8.38	0.133

*SLE* systemic lupus erythematosus, *BMI* body mass index, *WBC* white blood cell, *RBC* red blood cell, *HGB* hemoglobin, *RDW* red blood cell distribution width, *PLT* platelet, *LYM* lymphocyte, *NEU* neutrophil, *MON* monocyte, *ESR* erythrocyte sedimentation rate, *ALB* albumin, *A/G* albumin/globulin, *TG* triglyceride, *Cho* cholesterol, *HDL-C* high-density lipoprotein cholesterol, *LDL-C* low-density lipoprotein cholesterol, *eGFR* estimated glomerular filtration rate, *UA* uric acid, *C3* complement C3, *ANA* antinuclear antibody, anti-dsDNA, anti-double-stranded DNA antibodies, *SPISE* single-point insulin sensitivity estimator, *TAT* total adipose tissue, *VAT* visceral adipose tissue, *SAT* subcutaneous adipose tissue, *IMAT* intermuscular adipose tissue, *SMI* skeletal muscle index

### Body composition measurements in SLE patients with normal renal function and decreased renal function

In institution A, the TAT area (206.16 ± 138.27 vs. 240.22 ± 131.74, *p* = 0.006), VAT/TAT ratio (35.32 ± 12.46 vs. 42.18 ± 11.61, *p* < 0.001), SAT attenuation value (−95.34 ± 13.69 vs. −90.40 ± 14.66, *p* = 0.002), VAT area (75.63 ± 61.22 vs. 101.94 ± 59.98, *p* < 0.001), and IMAT area (8.41 ± 6.56 vs. 11.34 ± 9.26, *p* < 0.001) were higher in SLE patients with decreased renal function than in those with normal renal function (Table 1). No significant differences were observed in SAT area (*p* = 0.312), VAT attenuation value (*p* = 0.403), IMAT attenuation value (*p * = 0.871), or SMI (*p* = 0.133) according to the renal function status. Consistent with the results in Institution A, in Institution B we also found that the VAT/TAT ratio (*p* = 0.049), SAT attenuation value (*p* = 0.001), and IMAT area (*p* = 0.021) were higher in SLE patients with decreased renal function than in those with normal renal function (Supplementary Table S3). However, no significant group differences were found in TAT area (*p* = 0.872) and VAT area (*p* = 0.407).

### IMAT area as risk factors for the development of decreased renal function in SLE patients

In institution A, univariate logistic regression analysis showed that among the body composition parameters, TAT area, VAT/TAT ratio, SAT attenuation value, VAT area, and IMAT area were significant risk factors for decreased renal function in SLE patients (*p* < 0.05). The TAT area (*r*_s_ = 0.71, *p* < 0.001) and VAT area (*r*_s_ = 0.73, *p* < 0.001) were excluded because of a strong correlation with the IMAT area (Supplementary Fig. S1), and the remaining body composition parameters were included in the multivariate logistic regression analysis. The results showed that after adjusting for age, disease duration, hypertension, systolic blood pressure, and diastolic blood pressure, both the SAT attenuation value and IMAT area were independent factors for decreased renal function in SLE patients (Table 2). This association remained significant even after further adjustment for other factors that influence decreased renal function.

**Table 2:** Univariate and multivariate logistic regression analyses of body composition parameters and decreased renal function in SLE patients from institution A

	Univariate analysis		Multivariate analysis			
Variables	OR (95% CI)	*p*	Model 1		Model 2	
			OR (95% CI)	*p*	OR (95% CI)	*p*
TAT area, cm^2^	1.00 (1.00, 1.00)	0.039				
VAT/TAT ratio	1.05 (1.03, 1.07)	< 0.001	1.02 (0.99, 1.04)	0.200	1.02 (0.99, 1.06)	0.233
SAT area, cm^2^	1.00 (1.00, 1.00)	0.450				
SAT attenuation value, HU	1.02 (1.01, 1.04)	0.004	1.04 (1.02, 1.07)	< 0.001	1.04 (1.01, 1.08)	0.010
VAT area, cm^2^	1.01 (1.00, 1.01)	< 0.001				
VAT attenuation value, HU	0.99 (0.98, 1.01)	0.409				
IMAT area, cm^2^	1.05 (1.02, 1.09)	0.002	1.05 (1.01, 1.09)	0.019	1.05 (1.01, 1.10)	0.033
IMAT attenuation value, HU	1.00 (0.97, 1.03)	0.870				
SMI, cm^2^/m^2^	1.02 (0.99, 1.05)	0.201				

Model 1: Adjustment for age, disease duration, hypertension, systolic blood pressure, diastolic blood pressure

Model 2: Adjustment for RBC, HGB, NEU, ALB, Cho, and UA in addition to the variables in Model 1

*TAT* total adipose tissue, *VAT* visceral adipose tissue, *SAT* subcutaneous adipose tissue, *IMAT* intermuscular adipose tissue, *SMI* skeletal muscle index, *RBC* red blood cell, *HGB* hemoglobin, *NEU* neutrophil, *ALB* albumin, *Cho* cholesterol, *UA* uric acid

In Institution B, although univariate logistic regression analyses showed that both SAT attenuation value and IMAT area were significant risk factors for the decreased renal function in SLE patients (*p* < 0.05), in multivariate logistic regression analyses, only IMAT consistently was an independent risk factor for the decreased renal function in SLE patients after adjusting for potential factors affecting renal function (Supplementary Table S4).

### Correlation of eGFR with abdominal CT-based body composition measurements in SLE patients

In institution A, correlation analysis was used to explore the relationship between eGFR and all the participants’ clinical indicators and body composition parameters. The results are shown in Supplementary Table S5 and Supplementary Fig. S2. To further explore the effect of body composition parameters on eGFR, we performed a multiple linear regression analysis of body composition parameters with eGFR (Table 3). The TAT area (*r*_s_ = 0.93, *p* < 0.001) was excluded owing to a strong correlation with the SAT area; the VAT area (*r*_s_ = 0.73, *p* < 0.001) was excluded owing to a strong correlation with the IMAT area; the IMAT attenuation value (*r*_s_ = 0.71, *p* < 0.001) and VAT attenuation value (*r*_s_ = 0.77, *p* < 0.001) were also excluded owing to a strong correlation with the SAT attenuation value (Supplementary Fig. S1). The results showed that SAT area (*β* = 0.15, *p* = 0.004), SAT attenuation value (*β* = −0.52, *p* = 0.003), and IMAT area (*β* = −1.36, *p* < 0.001) were significantly correlated with eGFR in SLE patients after adjusting for BMI, age, history of hypertension, and blood pressure. The correlation between IMAT area (*β* = −1.15, *p* = 0.005) and eGFR remained significant even after adjusting for RBC, HGB, ALB, TG, Cho, UA, and C3 affecting renal function.

**Table 3:** Dose-response relationship between computed tomography (CT)-based body composition parameters and estimated glomerular filtration rate (eGFR) in SLE patients from institution A

	Model 1		Model 2		Model 3	
Variables	*β*	*p*	*β*	*p*	*β*	*p*
VAT/TAT ratio, %	−1.14	< 0.001	−0.28	0.153	−0.22	0.363
SAT area, cm^2^	−0.07	0.086	0.15	0.004	0.12	0.057
SAT attenuation, HU	−0.63	0.001	−0.52	0.003	0.09	0.712
IMAT area, cm^2^	−1.25	0.002	−1.36	< 0.001	−1.15	0.005
SMI, cm^2^/m^2^	−0.05	0.849	0.50	0.130	−0.36	0.923

Model 1: unadjusted

Model 2: adjustment for age, BMI, disease duration, hypertension, systolic blood pressure, and diastolic blood pressure

Model 3: adjustment for RBC, HGB, ALB, TG, Cho, UA, C3 in addition to the variables in model 2

*eGFR* estimated glomerular filtration rate, *VAT* visceral adipose tissue, *SAT* subcutaneous adipose tissue, *IMAT* intermuscular adipose tissue, *SMI* skeletal muscle index, *BMI* body mass index, *RBC* red blood cell, HGB hemoglobin, *ALB* albumin, *TG* triglyceride, *Cho* cholesterol, *UA* uric acid, *C3* complement C3

To further test the reproducibility of the association between IMAT area and eGFR in SLE patients, we included eGFR-related body composition variables for SLE patients from institution B (Supplementary Fig. S3) in multiple linear regression analyses, and VAT area (*r*_s_ = 0.73, *p* < 0.001) was excluded because of a strong correlation with the VAT/TAT ratio. Results showed that the association between higher IMAT (*β* = −0.98, *p* = 0.049), SAT attenuation (*β* = −1.17, *p* < 0.001), and lower eGFR remained significant after adjusting for potential influences on renal function (Supplementary Table S6).

### Mediation analysis of the association between the IMAT area and eGFR

**Fig. 3:**
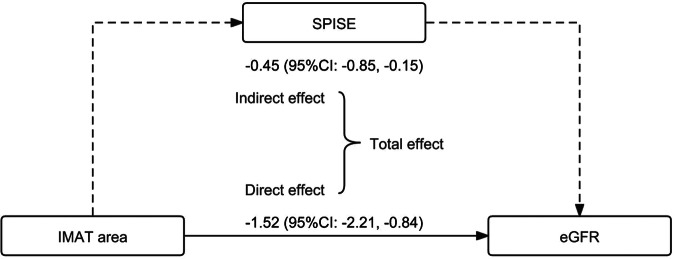
Mediation analysis of a single-point insulin sensitivity estimator (SPISE) demonstrating the association between intermuscular adipose tissue (IMAT area) and eGFR in SLE patients. Solid arrows indicate the direct effect of IMAT area on eGFR in SLE patients, and dashed lines indicate the indirect effect of IMAT area on eGFR in SLE patients via the mediator SPISE. Numbers indicate the β-values and their 95% confidence intervals for direct or indirect effects. SPISE, single-point insulin sensitivity estimator; IMAT, intermuscular adipose tissue; eGFR, estimated glomerular filtration rate; SLE, systemic lupus erythematosus

The relationship between SPISE and eGFR in SLE patients from institution A is shown in Supplementary Table S7. Even after adjusting for other factors affecting eGFR in SLE patients (including IMAT area), high SPISE (*β* = 3.69, *p* = 0.022) remained significantly associated with high eGFR. This indicated that increased insulin sensitivity (decreased insulin resistance) was linearly associated with increased eGFR in SLE patients, and this association was independent of the IMAT area. Considering the associations between IMAT area, SPISE, and eGFR in SLE patients, we applied mediation analysis to the study population. The results demonstrated that SPISE mediated the association between IMAT area and eGFR in SLE patients, and the indirect effect accounted for 22.84% of the total effect, and the *β* value (95% CI) of the indirect effect was −0.45 (−0.85, −0.15) (Supplementary Table S8 and Fig. 3).

### Correlation between IMAT area and renal histopathology in patients with LN

To further explore the effect of body composition parameters on LN, a subgroup analysis was performed on 42 patients from institution A who had undergone renal puncture biopsy. As shown in Supplementary Table S9, body composition parameters were not significantly associated with AI of LN, whereas VAT/TAT ratio (*r*_s _= 0.38, *p* = 0.012), VAT area (*r*_s _= 0.36, *p* = 0.018), and IMAT area (*r*_s_ = 0.32, *p* = 0.036) were all significantly associated with CI of LN. Furthermore, age (*r*_s_ = 0.38, *p* = 0.031), disease duration (*r*_s_ = 0.32, *p* = 0.040), hypertension (*r*_s _= 0.43, *p* = 0.004), RBC (*r*_s_ = −0.47, *p* = 0.002), and HGB (*r*_s_ = −0.40, *p* = 0.009) were also associated with CI. We performed a multiple linear regression analysis, and the results, as shown in Table 4, indicated that IMAT area (*β* = 0.13, *p* = 0.039) was still significantly and positively associated with CI after adjusting for age, disease duration, hypertension, systolic pressure, RBC, and HGB.

**Table 4:** Dose-response relationship between CT-based body composition parameters and chronicity index (CI)

	Model 1		Model 2		Model 3	
Variables	*β*	*p*	*β*	*p*	*β*	*p*
VAT/TAT ratio, %	0.02	0.389	0.05	0.076	0.05	0.092
VAT area, cm^2^	0.01	0.261	0.002	0.794	0.01	0.233
IMAT area, cm^2^	0.12	0.052	0.16	0.013	0.13	0.039

Model 1: unadjusted

Model 2: adjustment for age, disease duration, hypertension, systolic blood pressure

Model 3: adjustment for RBC, and HGB in addition to the variables in Model 2

*CI* chronicity index, TAT total adipose tissue, *VAT* visceral adipose tissue, *IMAT* intermuscular adipose tissue, *RBC* red blood cell, *HGB* hemoglobin

## Discussion

In this study, we analyzed abdominal CT-based body composition measurements of SLE patients from two institutions to find which measures were most closely associated with decreased renal function in SLE patients. We showed that the IMAT area was an independent risk factor for decreased renal function in SLE patients. Our data demonstrated that high IMAT area was associated with a reduction of eGFR in SLE patients, and the association was reproducible in the external validation dataset, with SPISE as an indirect indicator of insulin resistance playing a partial mediating role in the relationship. In addition, we performed a subgroup analysis of patients with pathologically confirmed LN and found that a high IMAT area tended to suggest a higher CI.

To our knowledge, this study was the first to analyze the negative effects of IMAT (ectopic adipose tissue infiltration in skeletal muscle, existing next to muscle fibers and between fascicles in humans) on renal function in patients with SLE. This finding is consistent with previous studies in patients with chronic kidney disease [23, 24]. Increased IMAT is not only associated with increased fatigue and decreased physical activity in patients with SLE but is also positively correlated with elevated blood pressure and the accumulation of inflammatory markers in plasma [25]. In addition, IMAT alters the skeletal muscle microenvironment by secreting pro-inflammatory cytokines and increasing local free fatty acid concentrations, leading to muscle dysfunction and insulin resistance, ultimately resulting in renal structural changes [26–28]. The results of our mediation analysis confirmed that the effect of the IMAT area on eGFR in SLE patients was partly achieved by altering the patients’ SPISE, an indicator of insulin resistance, which was consistent with the insulin resistance pathway described above; however, this mediating effect accounted for only 22.84% of the total effect. In addition, our subgroup analysis of 42 LN patients with renal puncture pathology results showed that the IMAT area was consistently and significantly correlated with CI, further confirming that an elevated IMAT area can indirectly or directly lead to glomerulosclerosis, fibrous crescent formation, renal tubular atrophy, and renal interstitial fibrosis in patients with SLE through pathways such as insulin resistance, thereby resulting in decreased renal function.

In our study, muscle mass was expressed as skeletal muscle area normalized by height (SMI). Although several studies have reported that sarcopenia is a common complication of chronic kidney disease [29, 30], our results showed no significant between-group differences in SMI observed between SLE patients with normal renal function and those with decreased renal function. This may be explained by the fact that skeletal muscle mass in the general population often peaks in early adulthood and declines beyond 45 years of age, whereas SLE tends to have an early age of onset, and patients are often still in their young adulthood. This makes the loss of muscle mass less pronounced when SLE-induced renal damage [24]. Therefore, low muscle quality (i.e., a greater presence of adipose tissue infiltration) rather than low muscle quantity is a key factor contributing to decreased renal function in SLE patients.

Notably, we identified SAT attenuation value as an independent risk factor for decreased renal function in SLE patients from Institution A, but we failed to validate this result in Institution B. We also found that SAT attenuation values were significantly associated with eGFR in SLE patients from Institution B, which was contradictory to the results in Institution A. This suggested a lack of robustness in the role of SAT attenuation values in identifying decreased renal function in SLE and the association with eGFR. The discrepancy between the findings of the two institutions may be related to the presence of varying degrees of peripheral edema and anasarca affecting SAT attenuation values in SLE patients with reduced renal function. High SAT attenuation values have recently been identified as indicators of altered adipose tissue quality, function, and structure [31], which may indicate adipocyte atrophy with reduced SAT fat storage capacity or inflammatory fibrosis, ultimately leading to adipose tissue dysfunction. In vivo, lipolysis is a well-recognized risk factor for insulin resistance and cardiovascular disease and may contribute to decreased renal function in SLE patients [32, 33].

Compared with existing work, the strengths of our research include the following. First, one of the strengths of our study was the large patient sample. Secondly, we obtained all available body composition measurements, contributing to a comprehensive understanding of the association between different tissues and decreased renal function in SLE patients. Thirdly, we analyzed SLE patients from institution A and institution B separately to verify that the results we obtained were reproducible. Finally, few studies have explored the mechanisms of association between body composition traits and disease. In the present study, we used mediation analyses to demonstrate that the association between a higher IMAT and a lower eGFR in SLE patients was mediated in part by a reduction in the patients’ sensitivity to insulin (insulin resistance). We can use this to address low muscle quality in SLE patients through dietary management and exercise, or by targeting insulin resistance, ultimately leading to improved renal function in SLE patients.

Our study had several limitations. First, this was a cross-sectional study that failed to determine causality. Therefore, in the future, we will further follow up on the clinical outcomes of the participants to obtain longitudinal data for validation analysis. Secondly, we retrospectively recruited SLE patients with available CT scans at the two hospitals, therefore, selection bias might have been introduced, and some SLE patients who underwent abdominal CT scans were due to acute illnesses, such as abdominal pain, that might have an impact on the patient’s eGFR. Thirdly, artefacts may reduce the accuracy of our body composition segmentation. Edema caused by decreased renal function may lead to elevated attenuation values of VAT, SAT, IMAT, and SM in SLE patients. In axial CT images of severely obese patients at the third lumbar vertebral level, part of the SAT was outside the field of view, which led to an underestimation of their SAT area. In addition, we used SPISE to characterize patients’ sensitivity to insulin because fasting insulin levels could not be measured. However, this index was originally developed based on Caucasians, and its accuracy for Asians needs further validation. Finally, this was a study limited to the Chinese population, thus limiting the generalizability of the findings to other ethnic groups.

In conclusion, our study highlights the significance of body composition parameters in determining renal function in SLE patients. The IMAT area was identified as an independent risk factor for decreased renal function. Targeting insulin resistance and body composition may offer potential strategies for protecting renal function in SLE patients. However, further longitudinal studies are warranted to validate these findings.

### Supplementary information

ELECTRONIC SUPPLEMENTARY MATERIAL

## Data Availability

The datasets used and analyzed during the current study are available from the corresponding author upon reasonable request.

## Abbreviation

95% CI: 95% confidence intervals
AI: Activity index
ALB: Albumin
ANA: Antinuclear antibody
BMI: Body mass index
C3: Complement C3
Cho: Cholesterol
CI: Chronicity index
CKD-EPI: Chronic Kidney Disease Epidemiology Collaboration
CT: Computed tomography
eGFR: Estimated glomerular filtration rate
HDL-C: High-density lipoprotein cholesterol
HGB: Hemoglobin
IMAT: Intermuscular adipose tissue
LN: Lupus nephritis
OR: Odds ratios
RBC: Red blood cell
SAT: Subcutaneous adipose tissue
SLE: Systemic lupus erythematosus
SMI: Skeletal muscle index
SPISE: Single-point insulin sensitivity estimator
TAT: Total adipose tissue
TG: Triglyceride
UA: Uric acid
VAT: Visceral adipose tissue
