# Supplementary material for: Association between higher intermuscular adipose tissue and decreased renal function in patients with systemic lupus erythematosus mediated by insulin resistance
Source: Insights Imaging. 2024 Jun 18;15:144. doi: 10.1186/s13244-024-01722-8 (PMC11182995; doi:10.1186/s13244-024-01722-8)
Supplement: Supplementary file 1 — ELECTRONIC SUPPLEMENTARY MATERIAL [file 13244_2024_1722_MOESM1_ESM.pdf]

Intermuscular adipose tissue and decreased renal function in patients with systemic lupus erythematosus mediated by insulin resistance
ELECTRONIC SUPPLEMENTARY MATERIAL

Table S1 Baseline characteristics and body composition parameters of patients with lupus nephritis (LN) from institution A

Variables	Total (n=42)
Age, years	29.05±11.02
Sex	
Male, n (%)	8 (19.0)
Female, n (%)	34 (81.0)
Height, cm	163.10±7.62
Weight, kg	60.12±10.54
BMI (kg/m^2)	22.71±3.93
Disease duration, years	2.14±4.19
Glucocorticoid use, n (%)	23 (54.8)
Hypertension, n (%)	9 (21.4)
Systolic blood pressure, mmHg	134.88±19.22
Diastolic blood pressure, mmHg	83.50±14.56
WBC, 10^{9}g/L	5.90±3.36
RBC, 10^{9}g/L	3.89±0.63
HGB, g/L	110.97±16.29
RDW, %	13.86±1.33
PLT, 10^{9}g/L	228.56±105.71
LYM, 10^{9}g/L	1.34±0.86
NEU, 10^{9}g/L	3.95±2.86
MON, 10^{9}g/L	0.74±1.47
ESR, mm/h	48.64±34.37
ALB, g/L	30.42±7.26
A/G	2.05±5.10
TG, mmol/L	2.14±1.12
Cho, mmol/L	5.63±2.25
HDL-C, mmol/L	1.31±0.62
LDL-C, mmol/L	3.10±1.42
eGFR, ml/min/1.73m^2	48.64±34.37
UA, umol/L	435.57±152.09
C3, g/L	0.19±0.33
ANA, n (%)	42 (100.0)
anti-dsDNA, IU/mL	340.60±391.82
AI	6.45±3.61
CI	2.29±1.97
TAT area, cm^2	200.21±108.31
VAT/TAT ratio, %	35.73±11.73
SAT area, cm^2	129.03±79.62
SAT attenuation value, HU	-91.86±15.43
VAT area, cm^2	71.18±41.93
VAT attenuation value, HU	-79.71±13.04
IMAT area, cm^2	7.49±5.24

IMAT attenuation value, HU	-58.24±8.37
SMI, cm^2/m^2	40.55±7.05

Abbreviation: BMI, body mass index; WBC, white blood cell; RBC, red blood cell; HGB, hemoglobin; RDW, red blood cell distribution width; PLT, platelet; LYM, lymphocyte; NEU, neutrophil; MON, monocyte; ESR, erythrocyte sedimentation rate; ALB, albumin; A/G, albumin/ globulin; TG, triglyceride; Cho, cholesterol; HDL-C, high density lipoprotein cholesterol; LDL-C, low density lipoprotein cholesterol; eGFR, estimated glomerular filtration rate; UA, uric acid; C3, complement C3; ANA, antinuclear antibody; anti-dsDNA, anti-double-stranded DNA antibodies; TAT, total adipose tissue; VAT, visceral adipose tissue; SAT, subcutaneous adipose tissue; IMAT, intermuscular adipose tissue; SMI, skeletal muscle index.

Table S2 Baseline characteristics and body composition parameters of all systemic lupus erythematosus (SLE) patients

Variables	Institution A (n=339)	Institution B (n=114)	*P*
Age, years	37.26±16.55	40.54±21.08	0.165
Sex			0.844
Male, n (%)	41(12.1)	13 (11.4)	
Female, n (%)	298(87.9)	101 (88.6)	
Height, cm	161.30±9.53	161.42±8.00	0.499
Weight, kg	58.58±14.38	58.27±12.94	0.652
BMI(kg/m^2)	22.39±4.61	22.44±4.73	0.898
Disease duration, years	4.04±5.95	2.77±5.07	0.165
Glucocorticoid use, n (%)	221 (65.2)	62 (54.4)	0.039
Hypertension, n (%)	83 (24.5)	36 (31.6)	0.136
Systolic blood pressure, mmHg	127.77±23.16	126.25±21.46	0.535
Diastolic blood pressure, mmHg	79.29±14.97	80.79±14.40	0.344
WBC, 10^{9}g/L	6.49±4.55	6.30±5.87	0.388
RBC, 10^{9}g/L	3.72±0.77	3.58±0.78	0.202
HGB, g/L	107.42±22.66	103.78±23.95	0.157
RDW, %	14.76±3.14	18.26±11.86	0.110
PLT, 10^{9}g/L	194.49±110.35	165.77±104.60	0.014
LYM, 10^{9}g/L	1.49±2.94	1.25±0.72	0.748
NEU, 10^{9}g/L	4.97±5.98	4.24±3.19	0.511
MON, 10^{9}g/L	0.51±0.81	0.50±1.03	0.905
ESR, mm/h	46.40±36.28	28.56±25.63	<0.001
ALB, g/L	34.87±7.19	35.93±7.51	0.100
A/G	1.25±0.40	1.34±0.48	0.106
TG, mmol/L	2.14±7.58	1.68±1.06	0.505
Cho, mmol/L	4.73±2.35	4.28±1.72	0.024
HDL-C, mmol/L	1.16±0.45	1.09±0.43	0.143
LDL-C, mmol/L	2.65±1.20	2.37±1.28	0.023
eGFR, ml/min/1.73m^2	103.15±44.07	108.40±44.44	0.487
UA, umol/L	322.28±183.75	319.03±141.12	0.951
C3, g/L	0.78±1.75	0.77±0.33	<0.001
ANA, n (%)	301(88.8)	104 (91.2)	0.464
anti-dsDNA, IU/mL	317.19±430.75	201.05±321.61	0.012
TAT area, cm^2	216.51±137.03	209.66±120.60	0.840
VAT/TAT ratio, %	37.40±12.59	36.32±13.53	0.450
SAT area, cm^2	132.89±86.64	129.15±75.93	0.907
SAT attenuation value, HU	-93.84±14.16	-90.71±15.87	0.086
VAT area, cm^2	83.62±61.95	80.51±61.83	0.633
VAT attenuation value, HU	-81.81±13.54	-80.23±15.36	0.315

IMAT area, cm^2	9.30±7.59	9.57±7.83	0.849
IMAT attenuation value, HU	-60.40±7.87	-61.47±8.22	0.228
SMI, cm^2/m^2	38.54±8.68	37.89±6.55	0.851

Abbreviation: SLE, systemic lupus erythematosus; BMI, body mass index; WBC, white blood cell; RBC, red blood cell; HGB, hemoglobin; RDW, red blood cell distribution width; PLT, platelet; LYM, lymphocyte; NEU, neutrophil; MON, monocyte; ESR, erythrocyte sedimentation rate; ALB, albumin; A/G, albumin/ globulin; TG, triglyceride; Cho, cholesterol; HDL-C, high density lipoprotein cholesterol; LDL-C, low density lipoprotein cholesterol; eGFR, estimated glomerular filtration rate; UA, uric acid; C3, complement C3; ANA, antinuclear antibody; anti-dsDNA, anti-double-stranded DNA antibodies; TAT, total adipose tissue; VAT, visceral adipose tissue; SAT, subcutaneous adipose tissue; IMAT, intermuscular adipose tissue; SMI, skeletal muscle index.

Table S3 Baseline characteristics and body composition parameters of SLE patients from institution B according to renal function

Variables	Total(n=114)	eGFR≥90(n=80)	eGFR<90(n=34)	*P*
Age, years	40.54±21.08	36.64±20.53	49.74±19.72	0.003
Sex				0.227
Male, n (%)	13 (11.4)	11 (13.8)	2 (5.9)	
Female, n (%)	101 (88.6)	69 (86.3)	32 (94.1)	
Height, cm	161.42±8.00	161.59±8.42	160.91±6.68	0.588
Weight, kg	58.27±12.94	59.42±13.78	55.13±9.87	0.196
BMI, kg/m^2	22.44±4.73	22.73±5.00	21.47±3.62	0.517
Disease duration, years	2.77±5.07	2.27±4.41	3.95±6.27	0.246
Glucocorticoid use, n (%)	62 (54.4)	36 (45.0)	22 (64.7)	0.054
Hypertension, n (%)	36 (31.6)	16 (20.0)	20 (58.8)	<0.001
Systolic blood pressure, mmHg	126.25±21.46	121.18±19.90	138.18±20.49	<0.001
Diastolic blood pressure, mmHg	80.79±14.40	77.30±13.65	89.00±12.84	<0.001
WBC, 10^{9}g/L	6.30±5.87	6.41±6.67	6.05±3.41	0.812
RBC, 10^{9}g/L	3.58±0.78	3.79±0.69	3.09±0.76	<0.001
HGB, g/L	103.78±23.95	108.82±23.92	91.91±19.70	<0.001
RDW, %	18.26±11.86	17.38±10.20	20.35±15.04	0.089
PLT, 10^{9}g/L	165.77±104.60	177.91±101.26	137.21±108.26	0.017
LYM, 10^{9}g/L	1.25±0.72	1.29±0.73	1.14±0.69	0.215
NEU, 10^{9}g/L	4.24±3.19	4.11±3.26	4.53±3.05	0.441
MON, 10^{9}g/L	0.50±1.03	0.52±1.23	0.43±0.20	0.219
ESR, mm/h	28.56±25.63	31.22±26.59	22.16±22.28	0.075
ALB, g/L	35.93±7.51	36.77±7.88	33.97±6.24	0.013
A/G	1.34±0.48	1.36±0.47	1.30±0.52	0.596
TG, mmol/L	1.68±1.06	1.65±1.05	1.75±1.11	0.740
Cho, mmol/L	4.28±1.72	4.30±1.80	4.24±1.55	0.916
HDL-C, mmol/L	1.09±0.43	1.10±0.44	1.08±0.41	0.974
LDL-C, mmol/L	2.37±1.28	2.39±1.34	2.34±1.14	0.825
eGFR, ml/min/1.73m^2	108.40±44.44	130.11±31.00	57.32±25.12	<0.001
UA, umol/L	319.03±141.12	277.78±96.88	402.79±177.36	<0.001
C3, g/L	0.77±0.33	0.80±0.34	0.70±0.30	0.161
ANA, n (%)	104 (91.2)	73 (91.3)	31 (91.2)	0.296
anti-dsDNA, IU/mL	201.05±321.61	205.87±315.09	189.47±342.01	0.298
TAT area, cm^2	209.66±120.60	209.27±121.56	210.57±120.10	0.872
VAT/TAT ratio, %	36.32±13.53	34.69±12.91	40.13±14.36	0.049
SAT area, cm^2	129.15±75.93	132.08±77.14	122.26±73.67	0.631
SAT attenuation value, HU	-90.71±15.87	-94.37±13.55	-82.07±17.70	0.001
VAT area, cm^2	80.51±61.83	77.20±59.73	88.31±64.72	0.407

VAT attenuation value, HU	-80.23±15.36	-81.41±14.59	-77.46±16.96	0.210
IMAT area, cm^2	9.57±7.83	8.44±7.11	12.24±8.88	0.021
IMAT attenuation value, HU	-61.47±8.22	-62.47±7.96	-59.10±8.47	0.045
SMI, cm^2/m^2	37.89±6.55	37.62±6.14	38.74±7.76	0.542

Abbreviation: SLE, systemic lupus erythematosus; BMI, body mass index; WBC, white blood cell; RBC, red blood cell; HGB, hemoglobin; RDW, red blood cell distribution width; PLT, platelet; LYM, lymphocyte; NEU, neutrophil; MON, monocyte; ESR, erythrocyte sedimentation rate; ALB, albumin; A/G, albumin/ globulin; TG, triglyceride; Cho, cholesterol; HDL-C, high density lipoprotein cholesterol; LDL-C, low density lipoprotein cholesterol; eGFR, estimated glomerular filtration rate; UA, uric acid; C3, complement C3; ANA, antinuclear antibody; anti-dsDNA, anti-double-stranded DNA antibodies; TAT, total adipose tissue; VAT, visceral adipose tissue; SAT, subcutaneous adipose tissue; IMAT, intermuscular adipose tissue; SMI, skeletal muscle index.

Table S4 Univariate and multivariate logistic regression analyses of body composition parameters and decreased renal function in SLE patients from institution B

	Univariate analysis		Multivariate analysis			
			Model 1		Model 2	
Variables	OR（95%CI）	*P*	OR（95%CI）	*P*	OR（95%CI）	*P*
TAT area, cm^2	1.00 (1.00, 1.00)	0.958				
VAT/TAT ratio	1.03 (1.00, 1.06)	0.052				
SAT area, cm^2	1.00 (0.99, 1.00)	0.527				
SAT attenuation value, HU	1.05 (1.02, 1.08)	<0.001	1.04	0.099	1.06 (0.98, 1.16)	0.143
VAT area, cm^2	1.00 (1.00, 1.01)	0.376				
VAT attenuation value, HU	1.02 (0.99, 1.05)	0.209				
IMAT area, cm^2	1.06 (1.01, 1.12)	0.023	1.08	0.048	1.19 (1.03, 1.39)	0.021
IMAT attenuation value, HU	1.05 (1.00, 1.11)	0.048	1.07	0.205	1.13 (0.95, 1.33)	0.166
SMI, cm^2/m^2	1.03 (0.95, 1.11)	0.485				

Model 1: Adjustment for age, hypertension, systolic blood pressure, diastolic blood pressure.

Model 2: Adjustment for WBC, RBC, HGB, A/G, UA in addition to the variables in model 1.

Abbreviation: TAT, total adipose tissue; VAT, visceral adipose tissue; SAT, subcutaneous adipose tissue; IMAT, intermuscular adipose tissue; SMI, skeletal muscle index; WBC, white blood cell; RBC, red blood cell; HGB, hemoglobin; A/G, albumin/ globulin; UA, uric acid.

Table S5 Correlation of estimated glomerular filtration rate (eGFR) with clinical characteristics and body composition parameters in SLE patients from institution A

Variables	r_s	*P*
Age, years	-0.59	<0.001
Sex	-0.001	0.989
Height, cm	-0.05	0.416
Weight, kg	-0.27	<0.001
BMI (kg/m^2)	-0.31	<0.001
Disease duration, years	-0.20	<0.001
Hypertension, n (%)	-0.49	<0.001
Systolic blood pressure, mmHg	-0.50	<0.001
Diastolic blood pressure, mmHg	-0.37	<0.001
WBC, 10^9g/L	-0.06	0.308
RBC, 10^9g/L	0.34	<0.001
HGB, g/L	0.27	<0.001
RDW, %	-0.03	0.555
PLT, 10^9g/L	0.07	0.175
LYM, 10^9g/L	0.09	0.119
NEU, 10^9g/L	-0.11	0.054
MON, 10^9g/L	-0.02	0.701
ESR, mm/h	-0.08	0.130
ALB, g/L	0.26	<0.001
A/G	-0.05	0.384
TG, mmol/L	-0.23	<0.001
Cho, mmol/L	-0.20	<0.001
HDL-C, mmol/L	-0.11	0.053
LDL-C, mmol/L	-0.10	0.093
UA, umol/L	-0.46	<0.001
C3, g/L	0.15	0.023
ANA, n (%)	0.10	0.074
anti-dsDNA, IU/mL	0.09	0.122
TAT area, cm^2	-0.31	<0.001
VAT/TAT ratio, %	-0.39	<0.001
SAT area, cm^2	-0.18	0.001
SAT attenuation value, HU	-0.06	0.282
VAT area, cm^2	-0.42	<0.001
VAT attenuation value, HU	0.21	<0.001
IMAT area, cm^2	-0.42	<0.001
IMAT attenuation value, HU	0.18	0.001
SMI, cm^2/m^2	-0.11	0.041

Abbreviation: BMI, body mass index; WBC, white blood cell; RBC, red blood cell; HGB, hemoglobin; RDW, red blood cell distribution width; PLT, platelet; LYM, lymphocyte; NEU, neutrophil; MON, monocyte; ESR, erythrocyte sedimentation rate; ALB, albumin; A/G, albumin/ globulin; TG, triglyceride; Cho, cholesterol; HDL-C, high density lipoprotein

cholesterol; LDL-C, low density lipoprotein cholesterol; eGFR, estimated glomerular filtration rate; UA, uric acid; C3, complement C3; ANA, antinuclear antibody; anti-dsDNA, anti-double-stranded DNA antibodies; TAT, total adipose tissue; VAT, visceral adipose tissue; SAT, subcutaneous adipose tissue; IMAT, intermuscular adipose tissue; SMI, skeletal muscle index.

Table S6 Dose-response relationship between computed tomography (CT)-based body composition parameters and estimated glomerular filtration rate (eGFR) in SLE patients from institution B

	Model 1		Model 2		Model 3	
Variables	*β*	*P*	*β*	*P*	*β*	*P*
VAT/TAT ratio, %	-1.14	<0.001	-1.10	<0.001	-0.48	0.127
SAT attenuation, HU	-1.43	<0.001	-1.34	<0.001	-1.17	<0.001
IMAT area, cm^2	-1.72	<0.001	-1.17	0.013	-0.98	0.049

Model 1: Unadjusted.

Model 2: Adjustment for sex, hypertension.

Model 3: Adjustment for age, WBC, HGB, RDW, NEU, MON, ESR, ALB, A/G, UA, anti-dsDNA in addition to the variables in model 2.

Abbreviation: TAT, total adipose tissue; VAT, visceral adipose tissue; SAT, subcutaneous adipose tissue; IMAT, intermuscular adipose tissue; WBC, white blood cell; HGB, hemoglobin; RDW, red blood cell distribution width; NEU, neutrophil; MON, monocyte; ESR, erythrocyte sedimentation rate; ALB, albumin; A/G, albumin/ globulin; UA, uric acid; anti-dsDNA, anti-double-stranded DNA antibodies.

Table S7 Association between single-point insulin sensitivity estimator (SPISE) and eGFR in SLE patients

SPISE	*β*	95%CI	*P*
Model1	4.48	2.79, 6.17	<0.001
Model2	2.80	0.69, 4.92	0.009
Model3	3.69	0.54, 6.84	0.022

Model 1: Unadjusted. Model 2: Adjustment for age, BMI, disease duration, hypertension, systolic blood pressure, diastolic blood pressure. Model 3: Adjustment for RBC, HGB, ALB, TG, Cho, UA, C3, IMAT area in addition to the variables in model 2.

Abbreviation: BMI, body mass index; RBC, red blood cell; HGB, hemoglobin; ALB, albumin; TG, triglyceride; Cho, cholesterol; UA, uric acid; C3, complement C3; IMAT, intermuscular adipose tissue.

Table S8 Mediation analysis of SPISE on the relationship between intermuscular adipose tissue (IMAT area) and eGFR in SLE patients

	β	95%CI
Total effect	-1.97	-2.62, -1.32
Natural direct effect (NDE)	-1.52	-2.21, -0.84
Natural indirect effect (NIE)	-0.45	-0.85, -0.15
%Mediated	22.84	11.48, 32.39

Table S9 Correlation of activity index (AI), confidence interval (CI) with clinical characteristics and body composition parameters

Variables	r_{AI}	P_{AI}	r_{CI}	P_{CI}
Age, years	-0.11	0.475	0.33	0.031
Sex	-0.18	0.264	0.14	0.396
Height, cm	-0.01	0.956	0.08	0.628
Weight, kg	-0.14	0.391	0.08	0.634
BMI (kg/m^2)	-0.10	0.519	0.05	0.746
Disease duration, years	<0.001	0.999	0.32	0.040
Hypertension, n (%)	0.20	0.215	0.43	0.004
Systolic blood pressure, mmHg	0.21	0.181	0.32	0.036
Diastolic blood pressure, mmHg	0.24	0.129	0.23	0.150
WBC, 10^{9}g/L	<0.001	0.998	0.01	0.946
RBC, 10^{9}g/L	-0.24	0.128	-0.47	0.002
HGB, g/L	-0.22	0.162	-0.40	0.009
RDW, %	0.01	0.97	0.12	0.465
PLT, 10^{9}g/L	-0.17	0.293	-0.02	0.915
LYM, 10^{9}g/L	-0.15	0.369	-0.03	0.879
NEU, 10^{9}g/L	0.05	0.749	0.01	0.956
MON, 10^{9}g/L	-0.14	0.383	-0.26	0.100
ESR, mm/h	-0.14	0.401	0.18	0.287
ALB, g/L	0.01	0.936	0.04	0.806
A/G	0.11	0.497	0.01	0.978
TG, mmol/L	0.15	0.364	-0.09	0.612
Cho, mmol/L	0.06	0.741	0.06	0.718
HDL-C, mmol/L	-0.01	0.941	0.07	0.685
LDL-C, mmol/L	0.09	0.604	0.12	0.493
eGFR, ml/min/1.73m^2	-0.18	0.282	-0.56	<0.001
UA, umol/L	0.23	0.224	0.01	0.967
C3, g/L	0.02	0.924	0.13	0.528
anti-dsDNA, IU/mL	0.25	0.123	-0.10	0.559
TAT area, cm^2	0.08	0.623	0.22	0.165
VAT/TAT ratio, %	0.13	0.418	0.38	0.012
SAT area, cm^2	0.07	0.673	0.12	0.446
SAT attenuation value, HU	0.07	0.648	0.07	0.646
VAT area, cm^2	0.10	0.514	0.36	0.018
VAT attenuation value, HU	-0.04	0.797	-0.13	0.413
IMAT area, cm^2	-0.08	0.603	0.32	0.036
IMAT attenuation value, HU	0.20	0.212	-0.17	0.291
SMI, cm^2/m^2	-0.15	0.358	-0.13	0.416

Abbreviation: BMI, body mass index; WBC, white blood cell; RBC, red blood cell; HGB, hemoglobin; RDW, red blood cell distribution width; PLT, platelet; LYM, lymphocyte; NEU, neutrophil; MON, monocyte; ESR, erythrocyte sedimentation rate; ALB, albumin; A/G, albumin/ globulin; TG, triglyceride; Cho, cholesterol; HDL-C, high density lipoprotein cholesterol; LDL-C, low density lipoprotein cholesterol; eGFR, estimated glomerular filtration rate; UA, uric acid; C3, complement C3; ANA, antinuclear antibody; anti-dsDNA, anti-double-stranded DNA antibodies; TAT, total adipose tissue; VAT, visceral adipose tissue; SAT, subcutaneous adipose tissue; IMAT, intermuscular adipose tissue; SMI, skeletal muscle index.

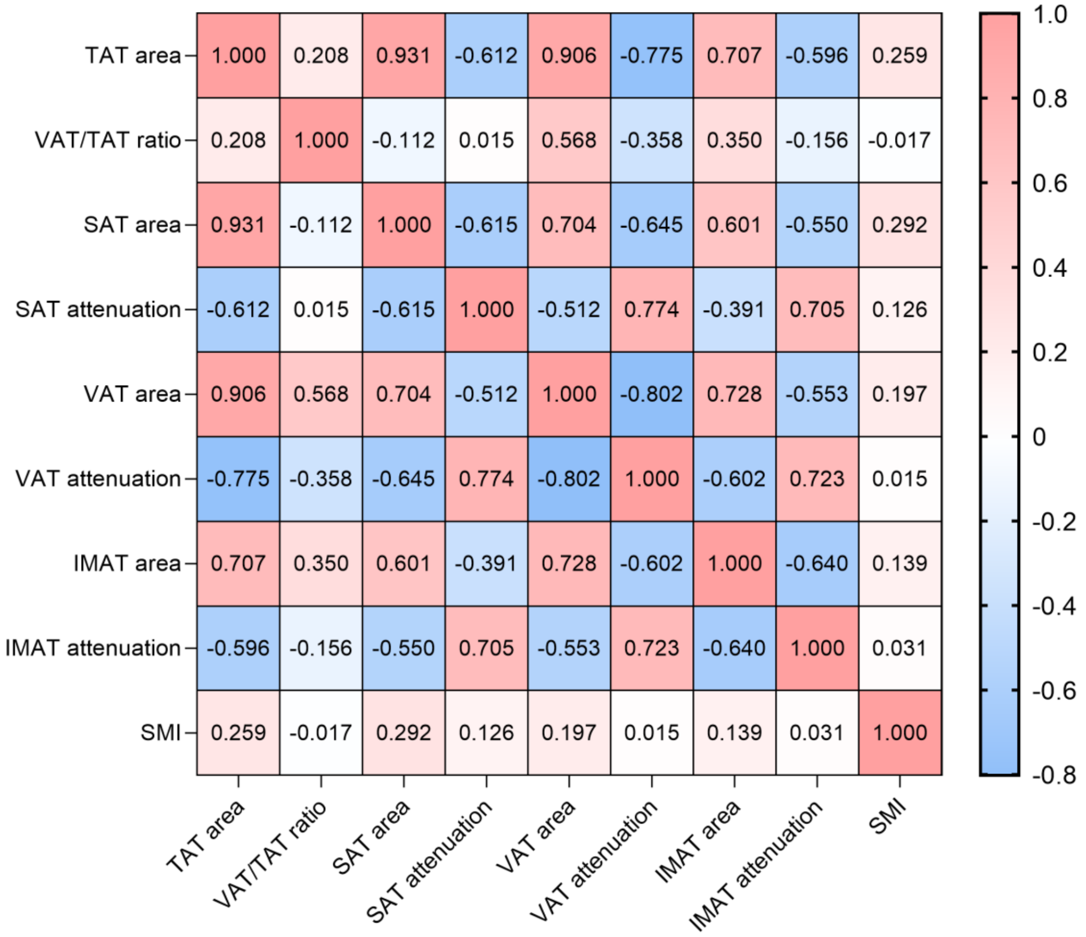

Figure S1 Correlations between body composition parameters in SLE patients from institution A

Abbreviation: TAT, total adipose tissue; VAT, visceral adipose tissue; SAT, subcutaneous adipose tissue; IMAT, intermuscular adipose tissue; SMI, skeletal muscle index.

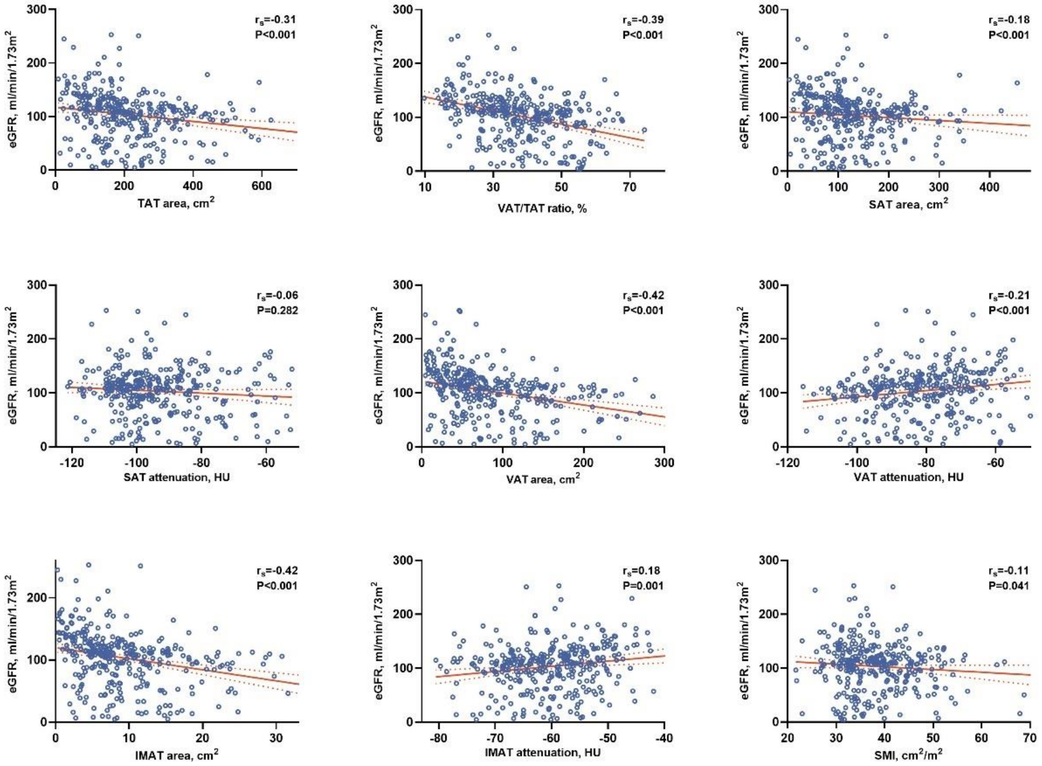

Figure S2 Correlation of body composition parameters with eGFR in SLE patients from institution A

Abbreviation: eGFR, estimated glomerular filtration rate; TAT, total adipose tissue; VAT, visceral adipose tissue; SAT, subcutaneous adipose tissue; IMAT, intermuscular adipose tissue; SMI, skeletal muscle index.

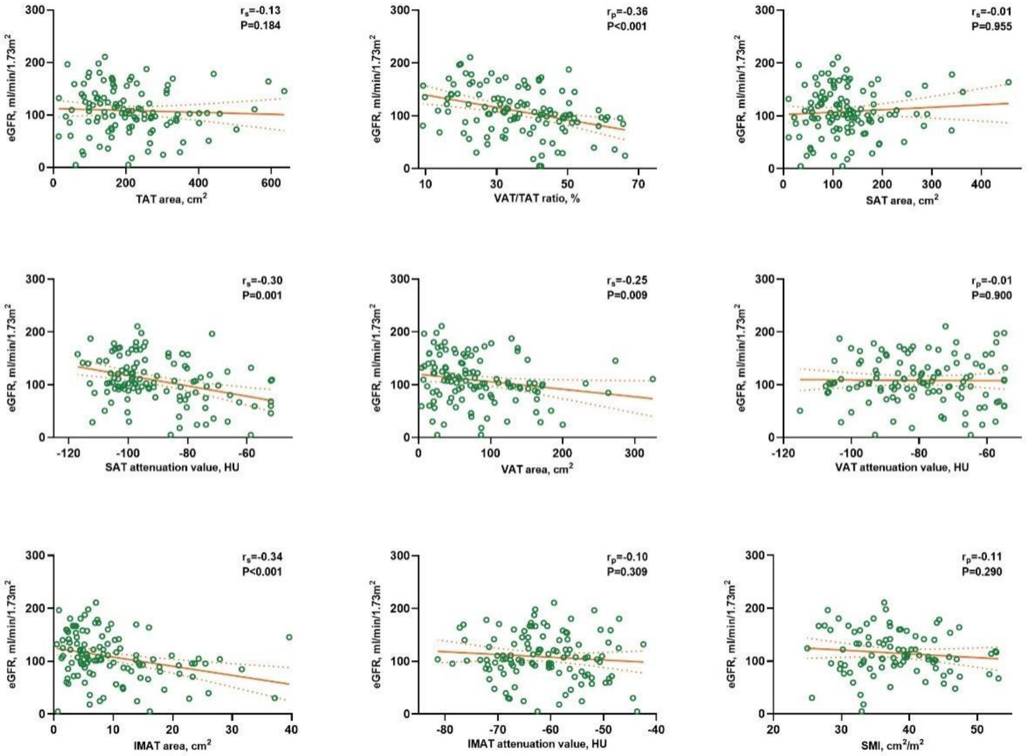

Figure S3 Correlation of body composition parameters with eGFR in SLE patients from institution B

Abbreviation: eGFR, estimated glomerular filtration rate; TAT, total adipose tissue; VAT, visceral adipose tissue; SAT, subcutaneous adipose tissue; IMAT, intermuscular adipose tissue; SMI, skeletal muscle index.
